# *Trypanosoma evansi*: A clinical, parasitological and immunological evaluation of trypanosomosis using a chronic rabbit model

**Published:** 2012-09-05

**Authors:** J.R. Ramírez-Iglesias, M.C. Eleizalde, E. Gómez-Piñeres, M. Mendoza

**Affiliations:** Universidad Nacional Experimental Simón Rodríguez, Instituto de Estudios Científicos y Tecnológicos (IDECYT), Centro de Estudios Biomédicos y Veterinarios (CEBIV), Apartado Postal 479245, Caracas 1041A, Venezuela

**Keywords:** IgG, Temperature, Total serum protein, Trypanosomosis, Weight

## Abstract

We evaluated the clinical, parasitological and immunological effects of a Venezuelan strain of *Trypanosoma evansi* (*T. evansi*) throughout in experimentally inoculated rabbits over the course of infection and compared them with the same aspect in healthy animals. Body temperature was recorded in degrees Celsius, animal weight in kilograms, serum proteins in g/dl using a refractometer, haematocrit percentage by capillary centrifugation and the anti-*T. evansi* IgG titer by indirect ELISA immunoassay, from both infected animals and controls for 95 days. Infected animals showed a higher body temperature, total serum protein and anti- *T. evansi* antibody titer, and a lower haematocrit and weight gain than controls. These differences were related to the presence of the parasites in the blood as detected micro-haematocrit centrifugation technique (MHCT) and direct microscopic examination (DME). This study confirms the usefulness of rabbits as a model for the study of trypanosomosis; the clinical features of the disease can be observed and the three characteristic stages, prepatent period, acute and chronic phase clearly defined over the course of the infection.

## Introduction

*Trypanosoma evansi* is mechanically transmitted by hematophagous insects (Tabanidae and Stomoxydae) and affects a number of tropical regions worldwide. This parasite causes the disease commonly known as “*Mal des Cadeiras*” in Brazil, “*Derrengadera*” in Venezuela (Desquesnes, 2004). In South America *T. evansi* principally affects equines although the infection of buffalos (Herrera *et al.*, 2004) and bovines (Gonzales *et al.*, 2003) has also been reported.

Over the course of infection, an initial prepatency period (PP) occurs between the inoculation of parasites in a healthy animal and their detection in the blood or tissue fluids, by direct microscopic observation. Following the PP, the disease progresses in two phases; an acute phase (AP), characterized by high levels of parasitemia and noticeable clinical symptoms, and a chronic phase (CP), characterized by low parasitemia which can either lead to emaciation or become clinically unapparent with undetectable changes in variables such as body temperature and haematocrit count (Fernández *et al.*, 2009).

The few experimental studies carried out on trypanosomosis caused by *T. evansi* have used horses (Marques *et al.*, 2000), goats (Dargantes *et al.*, 2005a, 2005b; Espinoza *et al.*, 2002), mice (Fernández *et al.*, 2009), dogs (Aquino *et al.*, 1999, 2002), cats (Da Silva *et al.*, 2009) and rabbits (Uche and Jones 1992; Uche *et al.*, 1992; Da Silva *et al.*, 2007; Ramirez *et al.*, 2011), etc., as experimental models. These studies have reported the characteristic clinical sign associated with the disease: high levels of parasitemia, a decrease in the haematocrit, weight loss, cellular infiltrations and the development of a marked humoral immune response (Uche and Jones 1992; Uche *et al.*, 1992). The aim of this study was to evaluate the characteristic clinical signs as well as parasitological and immunological aspects of trypanosomosis caused by a Venezuelan strain of *T. evansi* (TEVA1) using rabbits as an experimental model.

## Materials and Methods

### Laboratory animals

Five female New Zealand rabbits, weighting approximately 2 kg, were fed with commercial rations and water, available *ad libitum*. These animals were separated into two groups: two in the control group (C1 and C2) and three in the infected group (R1, R2 and R3). These last were inoculated intraperitoneally with 350 TEVA1 isolates parasites/animal following the protocol described by Ramirez *et al*. (2011).

### Sampling

The rabbits were sampled in the early morning at irregular intervals over a period of 95 days, by extracting about 2.5 ml of peripheral blood from the marginal vein in their ears. Each animal was evaluated 33 times during the course of the experiment. Infected rabbits were evaluated 5 times during the PP, 21 times during the AP and 7 times during the CP. Controls were sampled on the same days as the infected animals.

### Evaluation of temperature, haematocrit, total serum proteins and weight

For micro-haematocrit centrifugation technique (MHCT) analysis, 75 µl of extracted blood was taken with a heparinized capillary and centrifuged (3.000g, 10 min) to determine the haematocrit percentage. Serum was obtained from 2 ml of blood by centrifugation (2000g, 10 min) and total serum proteins (TSP) were calculated using a portable refractometer ATC F-302 LW Scientific^®^. The temperature and weight of the rabbits were measured using a rectal thermometer and a scale, respectively.

### Parasitological analysis

### Micro-haematocrit Centrifugation Technique (MHCT)

Capillary tubes were examined using a light microscope (10X objective), to detect motile trypanosomes near the buffy coat (Woo, 1970)

### Direct Microscopic Examination (DME)

Wet smears of 5 μl of extracted blood were examined by direct observation of 100 fields, using a phase contrast microscope (40X objective), to detect the presence of trypanosomes. Parasitemia (parasites/ml) was determined using the formula described by Brener (1962).

### Kinetic of anti-T. evansi specific antibodies over the course of the infection

The serological immune response to *T. evansi* was evaluated using sera obtained from blood samples extracted over the course of the infection. Specific IgG antibody production against the parasite was measured using an indirect enzyme-linked immunosorbent assay (iELISA). Polyvinyl 96-well plates (NUNC) were sensitized overnight in a humid chamber at 4 ºC with 100 μl/well of the clarified antigenic extract (20 μg/ml) in a 50 mM carbonate-bicarbonate buffer (pH 9.6). The protocol was performed following the method described by Ramirez *et al*. (2011). The optical density at 405 nm (OD_405_) was determined using an ELISA microplate reader (BioRad^®^ Model 3550). The OD_405_ readings obtained for the iELISA reflect the specific anti-*T. evansi* IgG titer. The cut-off value was established as the mean plus 3 times the standard deviation (X + 3 SD) of the OD_405_ reading from healthy rabbits (Ramirez *et al.*, 2011).

### Statistical analysis

The results of the parameters measured in the control group, during the experimentation (n = 66), were expressed as the mean ± standard deviation (X ± SD) and compared with the mean values for the infected animals during the infection, PP, and each phase of the disease (AP, CP), using the Student T-test. Differences were considered to be statistically significant at values of *P*<0.05.

## Results and Discussion

### Evaluation of the parasitemia and clinical parameters: temperature, haematocrit and weight during the course of infection

**Fig. 1:**
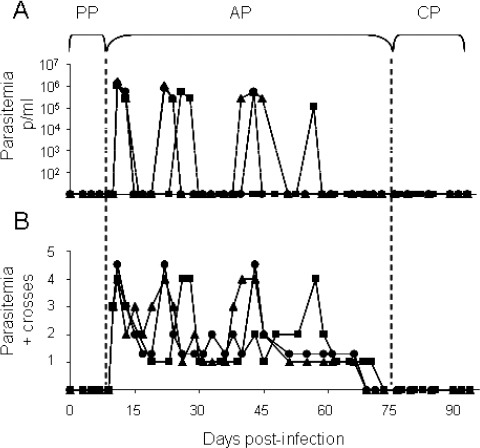
Evaluation of the parasitemia in rabbits infected with *T. evansi* over the course of the infection. Panel: (A) Parasitemia by DME (p/ml), (B) parasitemia by MHCT (crosses). (PP) Prepatency Period, (AP) Acute Phase and (CP) Chronic Phase. (•) R1, (▲) R2 and (■) R3.

The parasitemia as estimated by DME and MHCT for the three animals in the infected group (Fig. 1). During the first 9 days post-infection (PI), no parasites were detected in any of the infected rabbits showing that parasitemia levels were below the sensitivity thresholds of the two parasitological methods used.

**Fig. 2:**
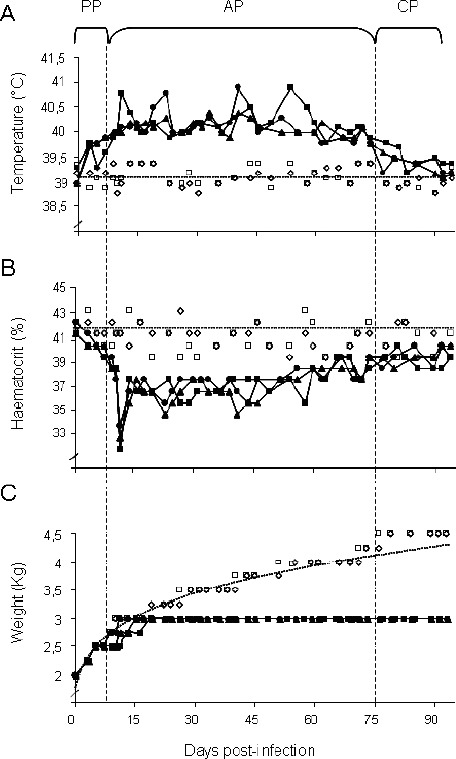
Evaluation of the clinical parameters in rabbits infected with *T. evansi* over the course of the infection. Panel: (A) Temperature (ºC), (B), Haematocrit (%) and (C) Body weight (Kg). (PP) Prepatency Period, (AP) Acute Phase and (CP) Chronic Phase. (•) R1, (▲) R2 and (■) R3; (◊) C1 and (□) C2 correspond to the values obtained for the three experimentally infected rabbits and the two healthy controls. The dotted lines (…..) in panels A and B indicate the mean values obtained for the healthy control group. The dotted plot (----) in C represents the increase in body weight in the healthy group during the experiment.

From day 10 PI, the presence of parasites in the blood of the infected animals was detected by MHCT, 1 day before observation by DME. The use of DME as a parasite detection method resulted in several false negatives between days 15 and 70, while MHCT detected the parasites in all the samples during this period. Three parasitemia peaks occurred in the three infected animals. The first peak reached maximum values of 1.8, 1.6 and 1.4 x 10^5^ parasites/ml on the same day, 11 PI, for the three infected animals, while the other two peaks registered lower values. The second peak reached values of 11.2, 8.4 x 10^4^ parasites/ml on day 22 PI for R1 and R2 respectively and 5.6 x 10^4^ parasites/ml on day 26 PI for R3. The third peak registered lower values of 5.6 x 10^4^ parasites/ml, on day 43 PI for R1 and R2, and 1.1 x 10^4^ parasites/ml on day 57 PI (Fig. 1A). The parasitemia thus showed an undulating behavior characteristic of the disease, and associated with the host immune response and the capacity of evasion of the parasite by antigenic. After the third parasitemia peak, from approximately 60 to 75 PI days until the end of the experiment, the parasitemia became undetectable by DME and MHCT. Based on these results we defined the three phases of the infection; (PP, AP and CP). The PP occurred between days 0 - 9 PI, during which no parasites were detected, the AP initiated after day 10 PI with the detection of the parasites and developed into the CP from day 75 PI when parasites were no longer detected by MHCT. Figure 2 shows the clinical parameters monitored: temperature (Fig. 2A), haematocrit (Fig. 2B), and weight (Fig. 2C) for the three animals in the infected group (R1, R2 and R3), and the controls (C1 and C2). During the PP, the means of the values of the clinical parameters measured in infected animals were not statistically different from the controls (P>0.05), 39.1±0.19 ºC for temperature and 41.5+0.9 % for haematocrit, and were also similar to those reported in the literature for healthy rabbits (Uche and Jones 1992; Da Silva *et al.*, 2007). The absence of clinical signs and undetectable parasitemias are characteristic of the PP for trypanosomosis caused by *T. evansi* (Aquino *et al.*, 1999; Marques *et al.*, 2000). The PP can vary depending on the animal model, the isolate, the quantity parasites inoculated, the inoculation route (Dargantes *et al.*, 2005b) and the parasite detection method used (Fernández *et al.*, 2009). In *T. evansi* experimental infections carried out in rabbits a PP of 7 days was observed after 4 × 10^5^ parasites were inoculated (Uche and Jones 1992). In this study, the delay of the appearance of the clinical signs and the low parasitemia can be attributed to the low inoculum levels, as well as the isolate used.

However, once the AP began, simultaneously with the detection of the parasites, the body temperatures and haematocrit of the infected rabbits underwent significant changes. After each parasitemia peak a marked decrease in the haematocrit and an increase in the body temperatures were observed (Fig. 2A and 2B). In this phase, the mean temperature of the infected rabbits was 40.2±0.24 ºC and statistically different to that of control group (*P*<0.05). Body temperatures never dropped below 40 ºC with a maximum of 40.9 ºC, from day 11 until day 60 PI (Fig 2A). The increase in the temperature of infected animals is a characteristic that has been previously described and related to the waves of parasitemia in experimental infections with *T. evansi* T. evansi (Uche and Jones, 1992; Aquino *et al.*, 1999; Marques *et al.*, 2000; Dargantes *et al.*, 2005b). The haematocrit was also significantly different between infected rabbits (38.05±1.94 %) and the controls (*P*<0.05). The most pronounced decrease in the haematocrit was observed on day 11 PI in all the infected animals, with minimum values of 34 %, 33% and 32%, for R1, R2 and R3, respectively (Fig. 2B). In infected individuals an inverse relationship between the haematocrit and parasitemia was observed.

Thus the haematocrit shows a marked decrease in infected individuals leading to an anemic condition when the parasitemia registers its maximum value. The destruction of erythrocytes is produced by phagocytosis as a consequence of the cellular damage caused by sialidase (Shehu *et al.*, 2006). After the third parasitemia peak, approximately day 60 PI, when parasites became undetectable by DME, the temperature and haematocrit started to return to the normal values registered for the healthy group. The recovery of the haematocrit following the descent of the parasitemia is due to the activation of erythropoyesis as has been described for hemolytic regenerative anemia (Da Silva *et al.*, 2009).

In the CP, from day 75 PI until the end of the experiment, no parasites were detected in the blood by either of the parasitological methods used. The clinical parameters: temperature (39.5±0.22 ºC) and haematocrit (39.6±0.93 %), returned to their normal values (*P*>0.05), approaching the mean values registered for the healthy group (Fig. 2A and 2B). This decrease in the parasitemia may be related to the immunological response of the host and the migration of the parasites to various tissues within the organism (Desquesnes, 2004).

Regarding body weight (Fig. 2C), the healthy control group showed a constant growth rate starting at 2 kg and increasing up to 3 kg in 12 days, reaching reached a maximum of 4.5 kg from day 90 until the end of the experiment, which represents the average weight for an adult New Zealand rabbit. Infected animals started to grow in the same way as the controls. However, a decrease in the growth rate observed after parasites were detected during AP, and the rabbits stopped gaining weight completely al around 20 days PI, registering final body weights that were statistically lower than those of the control (3 kg per rabbit), 1.5 kg below that registered for the control animals (4.5 kg) (*P*<0.05). Thus, infection by *T. evansi* produced a setback in the growth of the affected individuals compared to the healthy controls.

### Evaluation of the serum protein and the specific anti- T. evansi IgG titer over the course of the infection

**Fig. 3:**
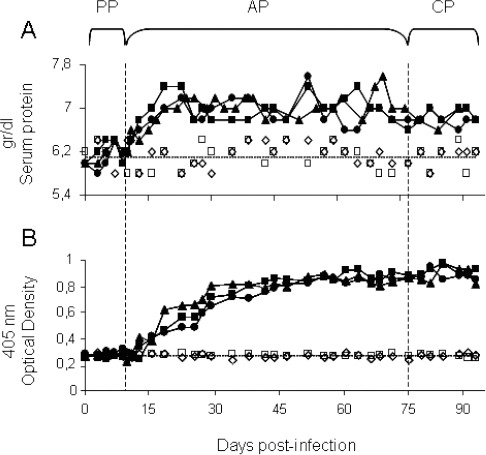
Evaluation of the serum proteins and the levels of IgG anti-*T. evansi* in infected rabbits over the course of the infection. Panel: (A) serum proteins (g/dl) and (B) OD_405_ readings. (PP) Prepatency Period, (AP) Acute Phase and (CP) Chronic Phase. (•) R1, (▲) R2 and (■) R3; (◊) C1 and (□) C2 correspond to the values obtained for the three experimentally infected rabbits and the two healthy controls, respectively, throughout the experiment. The dotted lines (…..) in panels A and B indicate the mean values obtained for the healthy control group.

In this study we investigated the pattern of specific humoral immune responses of rabbits infected with *T. evansi* during the experimental period. Figure 3 shows the values for the TSP and the OD_405_ registered over the course of the infection. In the PP the TSP values and OD_405_ readings of infected animals remained low, and statistically similar to those of healthy animals (6.2+0.21 g/dl, 0.32+0.03 respectively) (*P*>0.05). At the start of the AP, an increase in the TSP and OD_405_ was registered from days 11 and 13 PI respectively (Fig. 3A). The early increase in the TSP can be attributed to the increase in IgM antibody levels. This production of antibodies is a reflection of the evolution of the humoral immune response of the host towards the foreign agent. At the start of the infection, a primary humoral immune response develops against the parasite represented by IgM antibodies following by a secondary immune response, characterized by the synthesis of specific IgG. Thus, during the PP of the disease and at the beginning of the AP, the iELISA did not detect the presence of specific anti-*T. evansi* antibodies.

During the AP, after day 15 PI, the OD_405_ readings were always above the cut-off value for the sero-conversion (0.41), reaching values of around 0.9 from day 45 PI until the end of the experiment (Fig. 3B) and with an overall mean of 0.86±0.07, statistically different from the control group (*P*<0.05).

Similarly, after day 23 PI the TSP values remained high until the end of experiment, giving a mean of 6.9±0.28 (Fig. 3A), statistically higher than that of the controls (*P*<0.05). The increase in the levels of anti-*T. evansi* IgG produces a hypergammaglobulinemia, which has been described for different hosts infected with *T. evansi* (Aquino *et al.*, 2002; Da Silva *et al.*, 2007).

Overall, the simultaneous presence of IgM and IgG contributes to the increase in the TSP described. These antibody kinetics are similar to those reported from other studies, with the onset of detection of anti-*T. evansi* IgG by iELISA occurring between 10 - 23 days PI, together with the stabilization of antibody levels (Reyna-Bello *et al.*, 1998; Espinoza *et al.*, 2002).

During the CP, the iELISA detected the presence of anti-*T. evansi* antibodies even in the absence of clinical signs and at undetectable levels of parasitemia by the classical parasitological methods.

The maintenance of high levels of anti-*T. evansi* IgG for prolonged periods of time has been reported previously (Reyna-Bello *et al.*, 1998; Marques *et al.*, 2001), even after the disappearance of the parasite from the blood stream in animals submitted to chemo therapy (Monzon *et al.*, 2003). This demonstrates the limitations of the iELISA to distinguish between an active and a passive infection.

This study confirms the usefulness of rabbits as a chronic model for the study of trypanosomosis and permits the definition of the three characteristic stages (PP, AP and CP), as well as the observation of the clinical features of the disease, throughout the course of infection.
